# Ingredients to Mask the Aversive Taste of Medicines: Lessons from the Pharmaceutical and Food Industries and Home Remedies Adopted by Caregivers

**DOI:** 10.3390/foods15081413

**Published:** 2026-04-17

**Authors:** Susmita Paul, Okhee Yoo, Connie Locher, Lee Yong Lim

**Affiliations:** 1 Department of Pharmacy, School of Health and Clinical Sciences, The University of Western Australia, 35 Stirling Highway, Perth, WA 6009, Australia; susmita.paul@research.uwa.edu.au (S.P.); okhee.yoo@uwa.edu.au (O.Y.); connie.locher@uwa.edu.au (C.L.); 2 Centre for Optimisation of Medicines, School of Health and Clinical Sciences, The University of Western Australia, 35 Stirling Highway, Perth, WA 6009, Australia; 3 Institute for Paediatric Perioperative Excellence, The University of Western Australia, 35 Stirling Highway, Perth, WA 6009, Australia; 4 Wesfarmers Centre for Vaccines and Infectious Diseases, The Kids Research Institute, Nedlands, WA 6009, Australia

**Keywords:** aversive, taste, medicine, food, home remedy

## Abstract

Many approved oral paediatric medicines continue to have poor taste acceptance, suggesting that the ingredient blends employed in these medicines are not adequately effective in taste-masking drugs with strongly aversive tastes. To address this inadequacy, this narrative review provides a comparative evaluation of taste-masking ingredients used by the pharmaceutical industry with those employed in the food industry, as well as food items used by caregivers to mask the unpalatable taste of medicines for young children. Information was sourced from academic databases, industry publications, and caregiver forums on informal social platforms. Ingredients were classified into sweeteners, salts, acids, fats, peptides/amino acids, flavourants, cyclodextrins and polymers, with their taste-masking mechanisms delineated into receptor-level interactions and the creation of physical barriers and alternative dominant taste. Their applications are compared across the regulated medicinal and consumer food products, and in home remedies. Sweeteners show the highest cross-domain convergence as they are used in medicinal and food products and are recommended by caregivers. Peptides, amino acids, salt and texture modifiers applied in food and home remedies may have translational potential in medicines. Challenges, including drug–food interactions, regulatory constraints, and the need for combination approaches, are addressed. A decision framework is also designed to guide the development of simple, acceptable, and effective ingredient-based taste-masking systems for drugs with aversive tastes.

## 1. Introduction

The taste of an oral medicine is a major factor affecting treatment adherence in young children [1]. Many oral medicines contain drugs with aversive tastes. Studies have estimated that about 65% of drug molecules have a bitter taste, while others have sour or metallic tastes [2,3]. Drugs such as flucloxacillin, carbamazepine and amitriptyline also produce an offensive prolonged aftertaste [4,5]. Aside from the active pharmaceutical ingredient (API), some pharmaceutical excipients also have unpleasant tastes [6], including, ironically, the intense sweeteners saccharin, aspartame and cyclamate, which evoke unpleasant bitterness and metallic aftertastes [7]. Medicines having poor palatability are especially problematic when the medicines are to be taken at regular intervals over extended periods, as the unpleasant tastes can cause treatment cessation, leading to poor health outcomes and increased healthcare costs [8]. Conversely, access to better-tasting medicines has been linked to higher treatment completion rates [9].

The European Medicines Agency’s (EMA) Guideline on Pharmaceutical Development of Medicines for Paediatric Use, which was also adopted by the Australian Therapeutic Goods Administration (TGA), includes palatability and acceptability assessments in the medicine development plan [10]. The US Food and Drug Administration (FDA) also highlights “patient acceptability” (including palatability) as a critical component of drug products designed for the paediatric population [11,12]. In response, the pharmaceutical industry has expended resources to develop palatable oral medicines for children, and these taste-masking strategies have been reviewed extensively in recent publications [13,14].

Traditional taste-masking strategies employing a blend of sweeteners, flavouring agents and viscosity modifiers remain popular. In addition, physical barrier methods such as coating, microencapsulation, and granulation of drug substances with polymers, solid lipids and other materials, alongside chemical methods, such as drug complexation with carriers or ion exchange with resins, and the preparation of poorly water-soluble prodrugs and salt forms have been applied with varying degrees of success. More recent approaches include drug encapsulation in solid dispersions and nanocarriers. However, almost all taste-masking methods, when applied to child-appropriate formulations like oral liquids, chewable tablets or dispersible tablets, will invariably still require blending with sweeteners and flavouring agents to achieve taste acceptance of the final product [15,16]. Traditionally, sweeteners such as sucrose, sorbitol and sucralose and flavours such as strawberry, orange and bubble gum are incorporated into medicinal formulations to mask the bitter taste of drugs. Additionally, amino acids such as leucine and glycine, and citric acid, are used in combination with viscosity enhancers like glycerol and liquid sorbitol [17].

The aim of this narrative review is to identify and conduct a comparative evaluation of the range of ingredients applied by blending techniques alone (i.e., without the use of sophisticated equipment like a spray dryer) to moderate the taste of oral medicines. The review first provides an overview of the factors driving taste perception before exploring the ingredients used in approved oral medicines and novel bitter taste blockers developed by the pharmaceutical industry in recent years [13,18]. Many approved oral paediatric medicines continue to have poor taste acceptance, suggesting that the ingredient blends employed in these medicines are not adequately effective at taste-masking drugs with strongly aversive tastes [3,19]. To address this inadequacy, this review also evaluated strategies adopted by the food industry, where the use of ingredient blends to achieve desirable taste profiles is a well-honed and expertly applied technique. In addition, food items used by caregivers to mask the unpalatable taste of medicines for young children were also reviewed to provide additional insights when considered together with the taste-modifying ingredients used by the pharmaceutical and food industries.

The review focuses on direct blending techniques of the ingredients which, for oral medicines, offer advantages of formulation simplicity, cost-effectiveness, general applicability across multiple dosage forms, and accessibility in communities with limited resources. The blended ingredients may suppress the drug’s undesirable taste or enhance other flavours to modify the dominant taste perception of the medicine. By applying specific blending techniques, it may also be possible for the ingredients to provide a matrix that hinders drug access to taste receptors, thereby minimising drug–receptor interactions and the perception of the drugs’ aversive tastes [6].

## 2. Data Search and Collection Methodology

To identify the relevant literature for this review, a comprehensive search was conducted using the following electronic databases: PubMed, Medline, EBSCOHost and OneSearch (online search engine of the University of Western Australia Library). The search strategy used the following keywords and combinations thereof: ‘taste-masking’, ‘food industry’, ‘pharmaceutical industry’. The search strategy for OneSearch also applied the word ‘taste’ under its subject filter to enhance relevancy. Only peer-reviewed journal articles published in English were included, with no restrictions on publication year. A total of 646 articles were identified, of which 261 were excluded as duplicates. The remaining 385 articles underwent title and abstract screening. Of these, 84 articles that focused on ingredients reported to be responsible for taste-masking through simple blending techniques only were selected for full-text review. An additional 15 references were consulted on the basis of these 84 primary articles to better understand the mechanism of action of each ingredient category.

Grey literature such as government reports, patents, newsletters, and the websites of paediatric hospitals were also considered to ensure up-to-date information and alternative perspectives were collected. The collective information was synthesised and stratified into different food groups for discussion in this review.

To access the range of food items used in home remedies by caregivers, publicly accessible blog posts and expert commentaries were retrieved and analysed. Much of this information is not contained within peer-reviewed literature but is available on paediatric hospital pharmacy websites and caregiver forums on social media. The sources were identified via targeted Google searches using the following search phrases: ‘taste masking tips for pediatric medicine’ and ‘home remedies to modify the taste of bitter medicine’. Content published between 2015 and 2026 on English-language platforms were reviewed. Caregivers’ discussions on strategies they employ to encourage a child’s adherence to a prescribed medicine regimen were reviewed to obtain information on implementation practicality and effectiveness, as well as relevant child feedback. As the effectiveness of these home remedies is yet to be confirmed through formal taste evaluations, the credibility of the home remedies included in this review was appraised informally by cross-checking claims reported in at least two independent sources that described the same strategy.

## 3. Taste Perception

**Figure 1:**
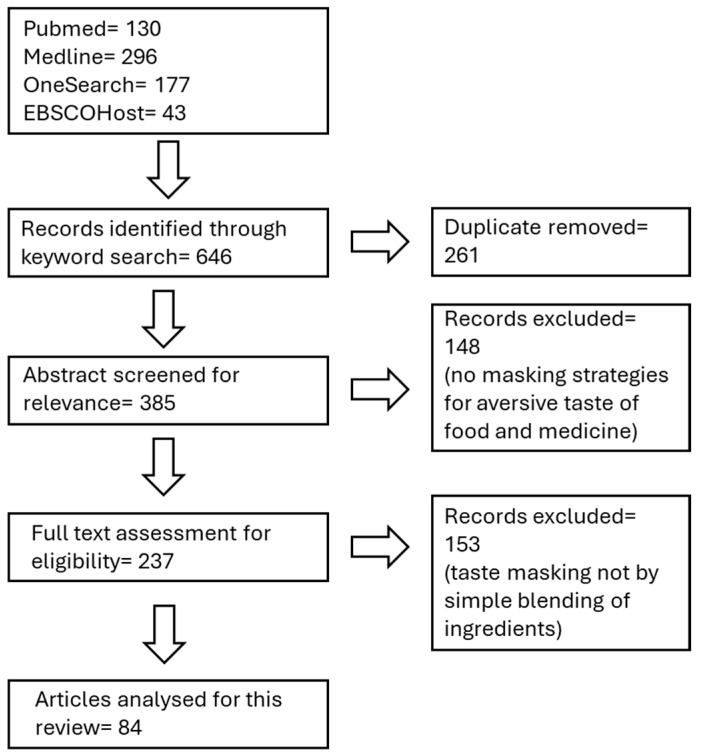
Flow diagram adapted from PRISMA for the search procedure of the published literature on taste-masking ingredients applied by simple blending techniques in food products and oral medicines extracted for this review.

Taste perception begins when molecules present in saliva bind with receptors on the surface of taste receptor cells in taste buds. Taste buds are located primarily on the dorsal surface of the tongue [20], although they are also expressed in the lower gastrointestinal tract (GIT) and other organs (respiratory, nervous, and reproductive) [21,22]. There are three main cell types in the taste buds (Figure 1) [23]. Type I cells are glial-like cells that provide support and protection essential for the proper functioning of the other cell types in the taste bud. Type II are G-protein-coupled taste receptor cells. Binding of a molecule to a taste receptor causes the release of adenosine triphosphate (ATP) that in turn excites the gustatory sensory afferents to send signals to the brain [24]. Type III are pre-synaptic neuron-like cells that may also be excited by ATP.

The five basic taste perceptions are bitterness, sweetness, sourness, saltiness and umami. The food industry also recognises other ‘secondary’ tastes, such as fatty, metallic, astringent and kokumi tastes [25]. Sweetness, bitterness and umami taste are detected by the Type II T1R and T2R families of receptors. Bitterness is mostly mediated by the T2R family which comprises 25–30 receptor types in humans; however, a bitter taste may be detected by as many as 80 receptor cell types [26]. Sourness is perceived by Type III proton-sensitive ion channels, whereas saltiness may be detected by Type I sodium-sensitive channels and potentially also by Type III cells. The transduction mechanisms for detecting fatty and kokumi tastes have not been fully elucidated but are likely mediated by subsets of the Type II cells.

**Figure 2:**
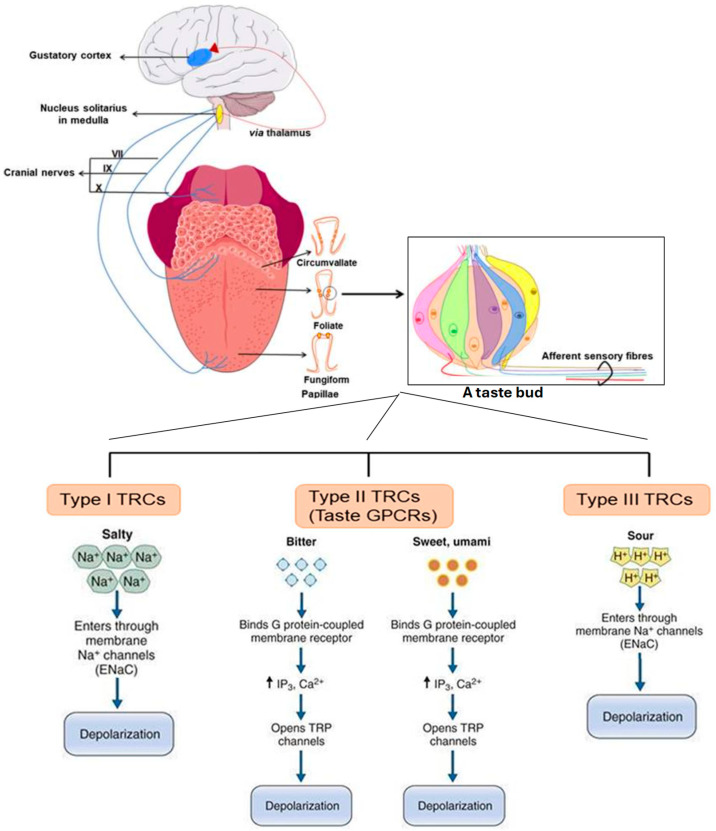
Anatomy and signal transduction pathways of taste perception: from tongue to brain [23].

Earlier models suggest that the tongue is topographically organised into different categories of taste buds, whereby bitterness is detected towards the back of the tongue and palate, sourness is detected along the edges of the tongue and palate, sweetness and umami are recognised on the tongue tip, and saltiness on the dorsum anterior [27] (Figure 2). This model is now recognised to be inadequate as taste buds for specific taste perceptions have been found distributed in all regions of the tongue, and a taste bud often contains receptor cells for a variety of taste sensations [26]. Moreover, sensory fibres innervating the taste buds each make synaptic contact with several taste receptor cells, and the stimulation of one type of taste receptor cell may influence the stimulation of other taste receptor cells through electrical interactions. The anatomy and physiology of the taste buds and sensory fibres, as well as their interconnectedness, suggest that the final taste perception is likely to be identified only after the sensory nerve signals are processed in the gustatory area of the brain stem. More research is needed to ascertain the mechanisms of cross-interaction; it is important that we also address other knowledge gaps at the molecular level, including the structure of taste receptors and their conformational changes after binding with taste molecules [28].

Despite the mentioned knowledge gaps, taste is widely recognised to be a complex form of multisensory perception. Apart from the signalling pathways of different taste receptors interacting with one other, some molecules can activate multiple taste receptors, e.g., γ-glutamyl peptides can simultaneously activate the CaSR receptor that imparts kokumi taste perception and the T1R receptors to enhance sweetness and umami tastes [25]. A taste receptor may also bind with molecules of diverse chemistry, and some molecules can alter the conformation of a taste receptor that then influences the receptor binding affinity with a different molecule. Moreover, the taste of some molecules is concentration-dependent, e.g., sodium chloride tastes pleasantly salty at low concentrations but has an aversive taste at high concentrations when it stimulates the PKD2L1 (sourness) and TR2 (bitterness) receptors [29].

Taste acceptance is further influenced by odour, texture, and mouthfeel [30]. Odour is detected by olfactory receptors in the nasal cavity when volatile substances released during chewing travel up the back of the mouth into the nose. Texture and mouthfeel refer to the physical consistency of the consumed product, including the physical changes produced as the product is chewed into smaller pieces in the mouth. The perception of softness, crunchiness, grittiness, chewiness, dryness and thickness are detected by mechanoreceptors in the mouth and aided by auditory receptors in the ear. A medicine without aversive taste may still be unacceptable to young children if it has an unpleasant odour or texture [31].

Taste is also influenced by chemesthesis, the perceptions of pungency, warmth or coolness that are mediated by the TRPV1 channels in the nerves surrounding the taste buds [32]. Some sensations may be better accepted than others, e.g., the cooling sensation of dissolving menthol versus the pungency of wasabi and astringency of tea tannins. TRPV1 channels also sense temperature and carbonation, resulting in spicy food having greater pungency when presented hot, and sour food having higher acceptance when combined with effervescence. Odour, texture, mouthfeel and chemesthesis are essential quality parameters for food products [33], but these elements are not routinely measured for oral medicinal products, including those designed for paediatric patients.

## 4. Taste-Masking Strategies for Oral Paediatric Medicines

Taste perception is traditionally not considered by the pharmaceutical industry to be a critical quality attribute to be measured for an oral medicine. Despite the widespread adoption of taste-masking strategies, there is a significant number of paediatric medicines that still have unpleasant tastes. In particular, liquid medicines containing antibiotics, like azithromycin, clarithromycin, erythromycin, and norfloxacin are associated with high levels of bitterness [34]. A study found 62.5% (15/24) of assessed oral antibiotic liquid preparations to have mean taste scores below 2.5 (with the taste scale ranging from 1—‘totally repulsive’ to 5—‘really yummy’), with metronidazole compounded with the Ora-Sweet™ vehicle having the lowest taste score of only 0.3 [35]. The medicines evaluated in these taste trials had been formulated with sweeteners and flavouring agents; therefore, their low taste scores suggest that the effectiveness of the adopted taste-masking strategies was not verified by human taste evaluation.

Regulations enacted by the FDA and EMA [36] have led to change, as reflected by the increasing number of publications on the evaluation of taste-masking strategies for oral paediatric medicines in recent years. A comparison of two reviews published twenty years apart [13,17] indicates that coating, microencapsulation, granulation, inclusion complexation, incorporation into carrier matrices, ion exchange with resins, and water-insoluble prodrugs remain popular methods for taste-masking oral medicines.

For solid oral dosage forms such as tablets, coating with Eudragit EPO (EPO) can be effective [37,38]. EPO is a cationic polymer that is insoluble in saliva, allowing EPO-coated tablets to have negligible drug release in the oral cavity and complete drug release in the lower gastrointestinal tract when the EPO dissolves in the acidic gastric fluid. Coated tablets may, however, pose a choking risk to young children, for whom other dosage forms such as minitablets, chewable tablets, orodispersible tablets, and liquid preparations may be better suited. These child-appropriate formulations often require more complex taste-masking strategies involving coated granules, inclusion complexes and solid dispersions [13].

Rendering a bitter-tasting drug into micron-sized spherical granules with smooth surfaces can facilitate the application of a uniform coating, which is an effective taste-masking strategy [39]. However, when the coating material does not have an acceptable taste or texture, and many do not, additional taste-modulating ingredients will be required to prepare the final formulations. As an example, cefuroxime axetil was prepared as taste-masked granules via wet granulation with micronized silica gel and as solid dispersions with sucrose and stearic acid [40]. The granules and solid dispersions were then blended with sweeteners (xylitol, sucrose, aspartame and acesulfame potassium), a viscosity enhancer (xanthan gum) and flavouring agents to prepare final powders for reconstitution into oral suspensions.

An emerging strategy is the preparation of drug inclusion complexes with β- or γ-cyclodextrins to limit drug interaction with taste buds. This has been applied with mixed effectiveness to taste mask a range of drugs [41]. Success is more difficult to achieve for high-dose drugs, as the 1:1 molar ratio of drug–cyclodextrin required for complexation can lead to unacceptably high amounts of cyclodextrins required for taste-masked formulations. Additionally, sweeteners and other taste-masking ingredients are often still required when the free drug in equilibrium with the cyclodextrin-complexed drug has a strongly aversive taste at a low detection threshold. Table 1 provides examples of commercial paediatric medicines available in Australia (https://www.tga.gov.au/), with ingredients that can be assumed to be used for taste-masking.

## 5. Taste Modulation by the Food Industry

Compared to the pharmaceutical industry, the food industry employs taste-modifying strategies far more extensively and with a fundamentally different approach. Oral medicines often require taste-masking of a single active pharmaceutical ingredient. The medicines are prescribed by clinicians, and patients are obliged to take the medicines according to the prescribed schedule. The pharmaceutical quality attributes of a medicine, alongside its safety and efficacy, are of greater priorities than taste acceptance, the taste of the medicine raising concern only if it causes patients to refuse to take it when required. From a safety perspective, oral medicines, particularly those designed for paediatric use, are formulated to not have attractive tastes, thus discouraging misuse.

Food items, by contrast, contain many components which interact to give unique taste profiles, and they are selected by consumers based on personal preferences that are strongly influenced by the sensory characteristics of the food product. Consumer preferences are also influenced through marketing of the differentiation in taste, cost and health benefits of the food products. The food industry therefore does not only mask unattractive tastes but also creates innovative tastes through the skilful blending of ingredients to achieve a perceived deliciousness to capture market share and assure brand loyalty. While medicines are typically prepared with purified materials that meet stringent pharmaceutical excipient quality standards, the food industry may apply complex ingredients (e.g., fruit juices) and processing methods (e.g., caramelisation) to generate multiple complex molecules, the identity and concentration of which may not be known, to produce a target taste profile for a food product.

With the rising popularity of fortified and functional foods, the food industry has also adopted taste-masking strategies that target specific bioactives in these products, e.g., the bitterness of minerals and peptides, and the astringency of flavonoids, isoflavones, polyphenols, and terpenes [42]. These strategies bear similarities to those more commonly associated with the pharmaceutical industry, and they include the following: (1) applying coatings of fatty acids and polymers to taste mask the astringency of tannic acid [43]; (2) complexation with cyclodextrins to preserve the taste and aroma of labile ingredients and mask the bitterness and astringent tastes of other ingredients [44]; and (3) the encapsulation in micron- and nano-sized systems to stabilize flavouring agents, enhance the perception of select flavours, and control flavour release during chewing for baked products, powdered foods, beverages and other processed foods [45].

It is not within the scope of this review to compare the plethora of methods applied in both the food and pharmaceutical industries. The review will instead focus on the use of ingredient blends as a taste-modifying strategy in the two industries. Additionally, while the ingredients are present naturally in fresh, cooked and/or fermented foods, the review will focus on the use of the individual ingredients rather than the whole foods.

## 6. Ingredients for Taste Modulation by Blending: A Current Perspective from the Pharmaceutical and Food Industries

Ingredients for taste modulation may be added to a product by simple mixing or by specific blending techniques that may include heating followed by cooling or grinding. Ingredients added by simple mixing may change taste perception by blocking an offensive taste through antagonistic interaction with receptors, or replacing the offensive taste with a stronger perception of more attractive tastes through central cognitive interactions in the brain [6].

To block a bitter-tasting molecule, the antagonistic ingredient must act on the same taste receptor, which can be challenging to achieve as there are multiple G-protein coupled bitterness receptors, and some molecules can stimulate bitterness taste by also modulating ion channels [46]. Alternatively, an undesirable taste may be modified by imparting a more dominating attractive taste, such as increasing sweetness with sweeteners [47], balancing flavours through ingredient combinations, such as salts and umami peptides [25], changing textures by creating a creamier, viscous mouthfeel or carbonation [31], and/or transforming saliva into a viscous matrix by adding polymer or fat to impede molecule access to the taste receptors [48]. Some ingredients, upon specific blending techniques, will enact physical barriers to hinder a molecule from interacting with taste receptors in the mouth [49]. Examples of these include the following: dispersing the aversive tasting molecule in molten fats followed by cooling to entrap the molecule in a solid fatty matrix [50]; co-melting the molecule with sugar followed by cooling to form solid dispersions [51]; co-grinding the molecule with cyclodextrin to form inclusion complexes [52]; and co-dissolving the molecule with amphiphilic polymers or lipids to entrap the molecule in micellar-like structures [53]. Some ingredients may fit into both categories, e.g., sucrose activates the sweetness receptor to counter the taste of the molecule, and when co-melted with the molecule, sucrose may potentially be a carrier that limits the molecule’s access to the taste receptors.

The following sections will examine the different classes of ingredients used for taste modulation applications in more depth.

### 6.1. Sweeteners

Sweeteners are pivotal ingredients in many medicines and foods as the taste of sweetness is agreeable to most people and preferred by children. An enhanced sweet taste may also minimise the perception of unattractive tastes [54]. Sweetness is perceived primarily from the activation of the sweetness receptors, which can be mediated by a wide range of chemically different compounds, including natural sugars (glucose, fructose, sucrose, maltose), artificial sweeteners (e.g., saccharin, aspartame, cyclamate), as well as sweet-tasting amino acids (d-tryptophan, d-phenylalanine, d-serine) and proteins (monellin, thaumatin) [55].

Sucrose is the classic natural sweetener, and it is applied as sugar and syrups in medicines and foods. Sucrose activates the T1R2/T1R3 sweet taste receptor and, unlike many other sweeteners, it does not activate other taste receptors even at high concentrations. However, it may bind allosterically with the CaSR receptor associated with the kokumi sensation [56]. As such, sucrose has superb organoleptic properties, producing sweetness with immediate onset and no lingering off-taste, with thick sugary syrups also imparting a favourable mouthfeel. Sucrose therefore sets a standard against which other sweeteners are evaluated. Sucrose is effective at reducing mild to moderate bitterness taste of molecules like urea, caffeine, propylthiouracil and quinine [57] but is ineffective in making strongly offensive tastes acceptable, as seen with currently available paediatric liquid formulations like flucloxacillin [58].

Artificial sweeteners include synthetic polyhydric alcohols or sugar alcohols, such as sorbitol, xylitol, maltitol, and mannitol [59]. These sugar alcohols are non-cariogenic and have lower caloric content than sucrose, making them popular sweeteners in food items and oral medicines. However, except for xylitol, which is as sweet as sucrose, the sugar alcohols taste less sweet per weight than sucrose. Some sugar alcohols like mannitol are popular ingredients in chewable and oral dispersible tablets because the crystalline mannitol undergoes endothermic dissolution in saliva to produce a pleasing cooling sensation that may help to mask aversive drug tastes [60].

Other non-caloric sweeteners have been developed in response to demands for safer ‘sugar-free’ products from consumers and governments facing the burdens of diseases linked to excess weight and obesity. Intense sweeteners like saccharin, aspartame, sucralose, and neohesperidin are 200 to 1500 times sweeter than sucrose [61], allowing their use at such low amounts in food and medicines as to be considered as non-caloric sweeteners [62]. Artificial sweeteners may be used alone; however, many have a lingering bitter aftertaste that requires blending with other sweeteners to confer a more desirable organoleptic experience [63,64]. As examples, saccharin and cyclamate are intense sweeteners that also activate the bitterness receptors [65,66]. When consumed together, cyclamate blocks the bitterness receptors (TAS2R31 and TAS2R43) that saccharin stimulates, while saccharin inhibits the bitterness receptors (TAS2R1 and TAS2R38) that cyclamate activates, and the result is a sweetness perception with reduced bitter aftertaste [66]. Another effective example of using mixtures is the combination of neohesperidin dihydrochalcone with γ-cyclodextrin, a cyclic oligosaccharide. This combination facilitates sweetness detection, reduces lingering aftertaste, and effectively masks the bitterness of paracetamol and quinine, as well as the astringency of green tea extract [67].

In recent years, with consumers demanding natural ingredients, artificial sweeteners are increasingly replaced by intense but low-calorie plant-derived sweeteners [68]. Examples include the steviol glycosides (from the leaves of *Stevia rebaudiana*)*,* monk fruit extract (from the flesh of Swingle or monk fruit), and thaumatin (from the flesh of Katemfe fruit). Rebaudioside A and mogroside V are the main sweeteners in the steviol glycosides and monk fruit extract, respectively. Like many artificial sweeteners, rebaudioside A and mogroside V stimulate the sweetness receptors as well as bitterness receptors, and they form synergistic blends with other sweeteners [69]. Thaumatin is a sweet-tasting protein [70] whose ability to reduce the bitterness of molecules may occur via direct action on the bitterness receptors or the modulation of signalling pathways [71]. Thaumatin is used in a wide range of food products [68], and its mixtures with other sweeteners have been found effective at masking the sourness of fruit juices and sodas [72]. Steviol glycosides are permitted sweeteners in medicines, while monk fruit extract is used in SuspendIt™, a commercial liquid vehicle for the compounding of oral medicinal suspensions for children [73].

Techniques other than simple blending have been applied to sweeteners in the food and pharmaceutical industries. Caramelisation applies heat to melt and oxidise sucrose to a brown-coloured product with desirable flavours and aromas. Caramelisation is a strategy that is widely applied in the food industry but is inappropriate for medicine manufacture as the heat input may affect drug stability. The pharmaceutical industry has also developed amorphous solid drug–sweetener dispersions by co-melting followed by cooling, or co-dissolution followed by solvent evaporation [74]. While these solid dispersions are currently aimed at increasing the in vivo dissolution of poorly water-soluble drugs, there may be potential for dispersions containing crystalline drug particles in a sucrose matrix for taste-masking applications.

### 6.2. Salts

The word ‘salt’ when used in the food industry refers to sodium chloride, a ubiquitous ingredient in savoury and sweet food products. Sodium chloride imparts a desirable saltiness taste to mask undesirable tastes (bitterness, metallic) as well as enhance other desirable tastes (sweetness, thickness, mouthfeel) [75,76]. The mechanisms underlying the varied sensory effects of sodium chloride are not well understood, except that it activates taste perception by both peripheral as well as central processing pathways [77,78]. The taste perception produced by sodium chloride is concentration-dependent. At concentrations of 10 mM (0.058% sodium chloride) or lower, sodium stimulates the saltiness receptor while also suppressing the activity of the TAS2R16 bitterness receptor [79] and enhancing the signalling of the T1R2/T1R3 sweetness receptors [47]. At higher concentrations (>300 mM or 1.74% sodium chloride), sodium produces undesirable bitterness and sourness perceptions by stimulating the PKD2L1 and TR2 receptors, respectively [29].

Unlike food products, oral medicines rarely contain sodium chloride for taste moderation. However, PCCA, a global supplier of excipients for medicine compounding, has recently recommended the addition of sodium chloride at 0.5–1% to mask the taste of extremely bitter drugs [80].

Several studies have shown the sodium ion to be the most effective cation for modifying bitterness taste [81,82,83]. In one study, sodium was found to be the most effective cation while glutamate and adenosine monophosphate (AMP) were the most successful anions in minimising the bitterness of pseudoephedrine, ranitidine, acetaminophen, quinine, and urea [82]. Sodium ions are likely to modify bitterness taste via peripheral interaction, as sodium chloride, sodium acetate and sodium gluconate have decreasing levels of saltiness but lower the bitterness of urea to comparable degrees [83]. Sodium acetate was shown to enhance the sweetness of a sweetener mixture when urea was present, but not when it was absent [75]. This suggests that sodium ions mainly reduce the bitterness of urea, allowing the sweetness to be perceived more strongly.

Potassium is often advocated as a substitute cation to reduce sodium intake; however, potassium chloride was found to be the only salt that failed to suppress the bitterness taste of urea in a study involving sodium chloride, lithium chloride, potassium chloride, sodium acetate and sodium gluconate [83].

Other sodium salts reported as taste modifiers include sodium homoeriodictyol, a flavanone extracted from *Eriodictyon californicum* which is effective at reducing the bitterness of guaifenesin, paracetamol, quinine and caffeine [84]. Another sodium salt with potential for taste-masking is sodium citrate. Sodium citrate is primarily applied to moderate pH and reduce drug oxidation in medicines and, like sodium chloride, it has salty and bitter tastes; however, sodium citrate also possesses a sour taste, the intensity of which can be varied by controlling the number of sodium ions attached to the citrate anion [85].

### 6.3. Acids

Organic acids, such as citric acid, lactic acid, acetic acid, tartaric acid and malic acid, are used as taste-modifying ingredients in the food industry, although the more common applications of organic acids in processed foods are as preservatives, pH regulators and antioxidants. Organic acids taste sour because they yield H^+^ ions upon dissociation, the sourness intensity being dependent on the H^+^ concentration. However, the chemistry of the anion also has significant influence [86]. In one study involving a sensory panel of 24 human participants, the perception of sourness of aqueous organic acid solutions (4% *w*/*v*, pH 3.6) decreased in the order of acetic (monocarboxylic, 60 g/mol) > malic (dicarboxylic, 134 g/mol) > tartaric (dicarboxylic, 150 g/mol) = lactic (monocarboxylic, 90 g/mol) > citric (tricarboxylic, 192 g/mol), suggesting that the sourness of the acids is not solely dependent on valency or molar concentration. Sourness perception occurs when H^+^ ions interact with ion channels like the PKD2L1 and OTOP1. Strong acids are fully ionised external to the taste receptor cell, generating H^+^ ions that interact with the H^+^-sensitive ion channels on the cell surface. Weak organic acids may generate the H^+^ ions intracellularly, after the undissociated acid molecules cross the phospholipid membrane into the taste receptor cell. Thus, weak organic acids may produce a stronger sourness taste if the acid is a relatively hydrophobic molecule [87].

Organic acids, apart from a common sourness taste, have characteristic taste and odour profiles. Acetic acid tastes and smells like vinegar, citric acid is reminiscent of citrus (lemon) juices, succinic acid elicits saltiness, bitterness and umami tastes, malic acid tastes like green apples, tartaric acid has a sharp and tangy taste, while lactic acid has a smooth and buttery taste. These unique tastes have been exploited in the food industry for taste modulation. Citric acid and malic acid are regularly added to augment the taste of lemon-flavoured candies and beverages while tartaric acid is effective at enhancing grape-flavoured foods and beverages. Citric, lactic, malic, and acetic acids are used to impart tartness, enhance sweetness, and stabilise the flavour profiles of food products [88]. Vinegar, malic acid, and citric acid are applied to reduce the perception of saltiness [89,90].

Organic acids also influence bitterness and sourness perceptions in a concentration-dependent manner: at concentrations below the detection threshold, the acids enhance bitterness; at moderate concentrations, they suppress bitterness and enhance sourness; and at high concentrations, they suppressed sourness but may enhance or diminish bitterness perception [91]. Additionally, organic acids modulate bitterness and sweetness responses in opposite ways, primarily by suppressing the responses of bitterness detecting neurons and reducing the bitter-compound-mediated repression of sweetness detection, thereby increasing the acceptance of bitter-tasting foods [92]. This process involves both a direct interaction of the acids at the taste receptor level and a broader sensory effect where the sharp, upfront sensation of sourness from the acids override the more persistent, lingering sensation of bitterness [92].

The pharmaceutical industry, like the food industry, also employs organic acids as preservatives, pH regulators and antioxidants. Additionally, organic acids (e.g., citric, tartaric, malic, and adipic acids) are used in combination with sodium bicarbonate to create effervescent medicinal formulations to provide a pleasant mouthfeel sensation that can help mask the taste of drugs like calcium carbonate [93]. Among the organic acids, citric acid is the most frequently used in the pharmaceutical industry, likely because of its favourable and familiar citrus aroma and flavour. Citric acid has been used in combination with sweeteners to mask the unpleasant taste of epinephrine, olopatadine, mirtazapine, and diclofenac in orally dispersing tablet formulations [94].

### 6.4. Peptides and Amino Acids

Oligopeptides and amino acids are increasingly employed as taste modifiers in the food industry to reduce dependence on sodium and sugar for enhancing the organoleptic properties of food products. The most established taste-modifier peptide is monosodium glutamate (MSG), a generally regarded as safe (GRAS) umami taste enhancer approved for food uses. MSG can suppress bitterness flavour [95] through antagonistic activity against the TAS2R16 receptor [96]. Combinations of MSG and nucleotides (e.g., IMP/GMP) exhibit strong umami synergy, significantly improving palatability and reducing perceived bitterness in protein hydrolysates [97]. MSG, however, has low acceptance following the publication of a misleading article in the New England Journal of Medicine in 1968, which described adverse symptoms later popularised as the “Chinese Restaurant Syndrome” [98].

Apart from MSG, a wide variety of peptides have been isolated from food sources or synthesized from amino acids for use as taste modifiers in the food industry [99]. Depending on their amino acid constitution, the peptides can give rise to salty, sourness, umami, sweetness, bitterness or kokumi tastes in foods and nutritional supplements. A number of food products have been identified to be rich in taste-imparting peptides. These include broad bean paste (sweet and bitter peptides), mushroom and chicken soup (umami peptides), and soybean curds (salty and kokumi peptides) [25]. Yeast extract, which is rich in taste peptides [100,101] and contains adenosine 5′-monophosphate, a known inhibitor of the transduction cascade of bitter taste receptors [102], is a potential bitter blocker. A powder developed from mushroom mycelia to block bitterness taste has been applied to foods and beverages containing coffee, cacao and chocolate [103].

Taste-modifying peptides have yet to be used to mask the tastes of medicines. However, a number of amino acids themselves also impart taste and are being explored as taste modifiers in medicines. Glycine (1–2% *w*/*v*) can reduce the bitterness of paracetamol in suspensions [104], while L-lysine successfully masked amlodipine bitterness in veterinary mini-tablets [105]. Similar to the taste-modifying peptides, the amino acids act through multiple mechanisms, with glycine and alanine imparting sweetness and potentially reduce bitterness taste via competitive receptor binding [106]; findings show that glutamate and aspartate enhance the umami taste [107] and L-arginine increases sweetness taste [108]. Amino acids are natural constituents of the body and are regarded to be safe for use in medicines. Amino acids such as alanine, lysine, arginine, glycine, aspartic acid, glutamic acid are already widely used in medicines to modify pH, solubilise poorly water drugs, or stabilize proteins for lyophilization. It is important to note that, when amino acids are used as taste modifiers, they have been found to not affect the other critical properties (e.g., pH) of the medicines.

### 6.5. Flavouring Agents

Flavouring agents are added in combination with sweeteners to achieve distinct favourable tastes [109]. This simple and convenient approach is widely applied (though not often validated) to mask the tastes of paediatric oral medicines like chewable tablets and liquid formulations. Flavouring of medicines is an art, with certain flavours identified and established through long experience of use to effectively mask specific aversive medicine tastes [110].

However, there is no consensus on universally preferred flavourings. A survey has found lemon-flavoured cough syrups are common in England and Australia, chamomile-flavoured herbal infusions are used for paediatric patients in Mexico, while bubble gum and cherry flavours are common in paediatric medicines in the United States [111]. Children are more likely to prefer flavours such as strawberry, bubble gum, cola and chocolate [112] and to dislike flavours like liquorice, peppermint and coconut [110,111]. The spicy flavour and anaesthetic properties of clove oil are also used for masking a range of bitter-tasting medicines [113]; however, some individuals will tolerate the pungent taste of cloves only if it is moderated with honey or vanilla flavour (e.g., as seen in paracetamol formulation) [113]. Likewise, menthol is not well tolerated by young children even though the cooling sensation produced by menthol dissolving in the mouth can numb the taste buds and retard bitter taste perception [114,115].

Flavouring agents applied by the pharmaceutical industry are predominantly chemically synthesized molecules of specific flavours presented as solutions or powders. The food industry on the other hand is more likely to use complex natural and processed ingredients to produce the desired taste profile for food products. A diverse array of flavouring ingredients is used in the food industry, ranging from whole foods like fruits, herbs, spices, allium vegetables and honey to processed ingredients like yeast extracts, sauces, oils, fats, butters and cheese. Flavouring may also be obtained by indirect means, e.g., adding the enzyme naringinase, to convert the bitter-tasting naringin present in citrus fruits to tasteless naringenin to improve the acceptance of orange juice [6]. The same technique is applied in wine, beer and soft drinks [116]. More recently, flavours encapsulated in liposomes have been deployed to more effectively mask the unpleasant oily tastes of ethyl caproate and isoamyl acetate in fermented beverages [117].

The principle of chemesthesis is often applied in the food industry. Basil, coriander, garlic, and ginger contain chemicals that activate the TRPA1 and TRPV1 ion channels [118] to produce pungent and spicy flavours effective at overpowering or balancing unsavoury tastes, while cinnamon’s naturally sweet, warm, and slightly spicy flavour effectively counteracts the bitterness perception of coffee. Combinations of ingredients, as seen for example in basil pesto (basil, garlic, olive oil, pine nuts, parmesan cheese) are also employed to impart desired flavours or reduce salt content in food [119].

The use of flavouring agents in foods and medicines is governed by regulations that provide definitions and labelling requirements for the different types of approved flavours. In Australia, the use and labelling of food flavours are governed by the Food Standards Australia New Zealand while flavouring agents in medicines are controlled by the TGA. Flavouring agents can be removed from approved lists when scientific findings raise safety concerns. As examples, the FDA in 2018 removed 6 synthetically derived flavours—benzophenone, ethyl acrylate, eugenyl methyl ether (methyl eugenol), myrcene, pulegone, and pyridine—from its approved food additives list when they were shown to cause cancer in laboratory animals. The EU Guideline on pharmaceutical development of medicines for paediatric use [120], which is adopted by TGA, further requires that the rationale for using a particular flavour in a paediatric preparation is clearly described and justified, and safety concerns, including the risks of allergies and sensitization, are addressed.

### 6.6. Fats

Raw fats have unpleasant tastes but foods where fats are blended with other ingredients may be perceived to have pleasantly rich creamy textures and tastes. There is growing evidence that free fatty acids (FFA) stimulate a ‘fat’ taste perception; however, because FFA are poorly soluble in saliva and there are lipases in saliva that break down other fat forms into FFA, the mechanism is not fully understood [121]. There are also significant individual variations in FFA perceptions that may be linked to eating behaviour and obesity. Notwithstanding this uncertainty, a traditional strategy commonly employed by the food industry to improve product taste is the inclusion of oils, butters and other fat-dense ingredients to impart rich taste and texture to subsume other unpleasant flavours and to form a thick matrix to slow the release of compounds with aversive tastes during chewing [122]. Fatty acids, fatty alcohols, waxes, and triglycerides are also used to form water-in-oil emulsions to encapsulate compounds with aversive tastes to enhance food palatability [123,124].

In the pharmaceutical industry, lipids have also been used to inhibit the interaction of aversive tasting drug molecules with taste receptors in the mouth. A recent comprehensive review of lipid-based strategies suggests that the aversive taste of drugs is effectively masked by coating the drug with a lipid either by solubilizing the drug in the lipid matrix or coating the drug with a lipid layer [125]. Almost all strategies reviewed applied technologies involving hot-melt extrusion and coating, spray drying and coating, spray congealing, melt granulation, or fabrication of colloidal systems like liposomes and solid micro/nanoparticles.

The technique of simply blending with lipid ingredients is less often reported for the taste-masking of drugs. However, the bitterness of midazolam, tramadol and prednisolone have been found to be effectively taste-masked by blending the respective drugs with molten cocoa butter in the form of chocolate together with other taste-modulating ingredients (sweetener and viscosity modifier) [126,127,128]. Taste-masking by simple blending of drugs and lipids may be facilitated through complexation that makes it difficult for the drug to interact with taste receptors. A study involving mixing the phosphate-buffered solutions of five bitter-tasting drugs and the sodium salts of eight fatty acids suggested that taste-masking was effective when specific drug and lipid molecules are able to spontaneously form an insoluble binary complex through hydrogen/ionic bond formation and hydrophobic interactions [129]. Taste-masking may also have been mediated by an increase in salivary viscosity, stimulated by fat, which hinders drug–receptor interactions and improves overall mouthfeel [17,130].

### 6.7. Polymers

A wide variety of polymers are used in medicinal product formulation for taste-masking purposes, and they have been the subject of several reviews [5,13,131]. The polymers include natural hydrocolloids (xanthan gum, carrageenan, pectin, gelatine, alginate), and synthetic polymers (polyvinyl alcohol, Eudragit E-100, ethylcellulose, hydroxypropylmethyl cellulose (HPMC)). Polymers are traditionally employed to create a physical barrier to prevent drug interaction with the taste receptors in the mouth, e.g., using gelatine to encapsulate the drug or using a polymer as coating for tablets and microparticles. Polymer coating of a solid dosage form is highly effective for taste-masking; however, it requires access to suitable coating equipment, and the coated tablets and capsules are designed to be swallowed, which may not be suitable for very young children prone to choking risks.

A more accessible approach is the blending of hydrocolloid solutions to modify the rheology of liquid medicines. Hydrocolloids like xanthan gum are popular ingredients in paediatric oral liquid medicines [13,132], their presence increasing the liquid viscosity to improve mouthfeel and hindering drug access to the taste receptors in the mouth [133]. Polymer–drug interactions further aid taste-masking by binding the drug molecules via hydrophobic interactions with the non-polar domains of the hydrocolloids or via hydrogen bonding or electrostatic binding with the hydrophilic domains of the hydrocolloids. Hydrocolloid solutions with good taste, e.g., gelatine, alginate and pectin, have also been cast into moulds to prepare chewable medicinal gummies [134,135].

Another simple technique employs amphiphilic block copolymers that dissolve in water to form micellar structures, allowing lipophilic drug molecules with aversive taste to partition into its core. A recent study evaluated several such water-soluble block copolymers [136] and showed that mPEG-PLLA (methoxy polyethylene glycol-poly (L-lactic acid)) at a concentration of 0.16% was most effective at masking the taste of berberine (0.05 mg/mL). mPEG-PLLA at 0.16% is above its critical micelle concentration (<10 µg/mL), forming micelles (mean size 264 nm) that solubilise berberine within its PLLA chains. Taste-masking, verified using human sensory evaluations, was attributed to the colloidal micelles being unable to interact with the taste receptors. Unlike the inclusion complexes formed with cyclodextrins which require the guest drug molecule to fit into the ring of the host molecule (Section 6.7), the amphiphilic micelles are less selective and may enable a more general application to all hydrophobic molecules. They may also be useful for liquid medicines containing a range of bitter bioactive molecules, as is commonly the case in multi-component traditional Chinese medicines.

Additionally, drug–polymer solid dispersions (or inclusion complexes) have been prepared by blending following by co-melting and cooling [137]. Like the sucrose–drug solid dispersions, these are currently aimed at modifying the oral bioavailability of drugs. The sustained-release inclusion complexes prepared with hydrophobic polymers [138] may, however, also have potential for taste-masking. Further studies to confirm this will be required.

Hydrocolloids are widely used to enhance the sensory properties of food products by modifying their rheology and texture [133]. Hydrocolloids such as xanthan gum, HPMC and guar gum are used as thickeners to impart the desired viscosity and mouthfeel to soups, gravies, salad dressings, sauces and toppings, while gelling agents like alginates, pectin, carrageenan, and gelatine are applied to create the desired textures in ice creams, jams, jellies, cakes and candies. The use of vegetable gums (gum Arabic, acacia gum) as texture-modifying ingredients to reduce bitterness taste is particularly attractive for functional beverages [133] while dextrans are effective at masking beany notes, bitter taste, and offensive aftertaste [17]. Nutritional bars prepared from bitter plant extracts (e.g., ginseng) have been taste-masked by adding gelatine or sodium alginate to the formula [139]. Apart from texture modification, the dissolution of the hydrocolloids during chewing may form a coat around the taste buds to minimize their stimulation by offending food components [13]. Hydrocolloids may also interact with other taste-modifying ingredients, e.g., hydroxypropyl cellulose, carboxymethyl cellulose, sodium alginate, and xanthan gum have been found to suppress the sourness of citric acid and the bitterness of caffeine while enhancing the sweetness of saccharin [140].

### 6.8. Cyclodextrin, Maltodextrin and Ion Exchange Resins

Cyclodextrins are cyclic polysaccharides that form inclusion complexes with drug molecules [63,141]. The inclusion complexes may be prepared by simple mixing of drug and cyclodextrin in solutions or co-grinding of the solid ingredients [142]. Complexation reduces taste perception as the number of free drug molecules available to interact with taste receptors is reduced and the drug-cyclodextrin complex, which houses the drug molecule within the cyclodextrin ring structure, is unable to bind to taste receptors.

The taste-masking efficiency of the inclusion complexes has been correlated to the association constant between drug and cyclodextrin, with studies showing that, while the tastes of fluoxetine [143], lumefantrine [144], ibuprofen [17] and captopril [145] have been successfully masked, the tastes of spironolactone [146] and cetirizine [147] have not. The association constant reflects how tightly the drug binds to the cyclodextrin. A high association constant may give rise to a stable complex that remains intact during transit in the mouth while a low association constant indicates a complex that dissociates easily in saliva to yield free drug and perceived bitterness. The choice of cyclodextrin matters, as the sweet-tasting β-cyclodextrin performed better than the α- and γ-cyclodextrins in the case of cetirizine [147]. Effective taste-masking requires a cyclodextrin–drug molar ratio of 1 or greater, as each cyclodextrin molecule can accommodate no more than 1 drug molecule. This limits applications to low-dose drugs in order to minimise the cyclodextrin concentration required in the final formulation.

Of the different cyclodextrins, β-cyclodextrin and its hydroxypropyl derivative are the most widely applied in medicinal formulations and, a wide variety of bitter-tasting drugs have been trialled [41]. Additionally, several commercial taste modifiers incorporating cyclodextrins are available. CAVAMAX^®^ [148] contains γ-cyclodextrin, which has a larger ring diameter than β-cyclodextrin, and is marketed for the compounding of taste-masked gummies of botanical bioactives. γ-Cyclodextrin in combination with neotame and mPEG-PLLA is also present in the Huanglian Jie-Du Decoction [149] while the Qingre Huazhi Decoction [150] contains *β*-cyclodextrin in combination with sucrose, sucralose, sweet orange fruit powder, citric acid, malic acid, and orange essence. Both decoctions are used to reduce the bitterness of traditional Chinese medicines.

Apart from cyclodextrins, high-amylose maltodextrin and ion exchange resin have also been used as complexation agents to mask the taste of drugs. High-amylose maltodextrin is effective in masking the taste of loperamide hydrochloride and dextromethorphan hydrobromide in paediatric syrup formulations [151] while oral dispersible minitablets of oseltamivir formulated with maltodextrin were found to have acceptable taste by a human sensory taste panel [152]. Liquid preparations of chlorpheniramine maleate, diphenhydramine HCl, ephedrine HCl, noscapine HCl, amphetamine sulphate, and orbifloxacin have been successfully taste-masked using ion-exchange resins [17].

In the food industry, since the 1970s, Japanese researchers have conducted studies into the use of cyclodextrin inclusion complexation to improve the taste of soybean, rice, casein, fish oil and citrus fruit products [142]. β-Cyclodextrins are also used, at a price comparable to microencapsulated flavours [44], to encapsulate highly volatile, light- and heat-sensitive flavour molecules to improve their handling, storage stability and aroma/flavour release rates to achieve the desired flavour profiles [153]. Maltodextrin is also widely used in the food industry as a taste-masking agent, having the ability to enhance or balance flavours and improve a product’s overall palatability and mouthfeel at a cost less than that of cyclodextrin [154]. As an example, the bitterness of enzyme-treated soy protein was found effectively masked in bread by the addition of maltodextrin [155].

### 6.9. Bitter Blockers

An emerging approach alternative to using taste-masking ingredients and supported by EMA guidelines is to employ a taste blocker. The principle is that, by blocking the bitter receptors, the final product will be neutral tasting, thus overcoming personal taste preferences and avoiding the creation of highly palatable medicines. A recent systematic review [18] evaluated 17 bitter blockers reported in the literature, and suggested that sodium acetate, sodium gluconate and adenosine 5′-monophosphate have good pharmaceutical ‘usability and safety profile’ based on their GRAS status and evidence of effectiveness in human sensory panels. These will need further exploration to determine their effectiveness as generic medicine taste-masking agents.

Several proprietary products are already marketed as bitter blockers. While their actual constituents remain undisclosed, the products appear to rely on taste peptides as bitter taste blocking components in combination with sweeteners and/or viscosity modifiers [156,157]. An example is Bitter-Bloc™, described as ‘an organic, water-soluble protein powder’ formulated to modulate bitter taste receptors [158]; however, no evidence for its use in medicines or foods could be found. PCCA has also developed several proprietary bases, including Bitter Drug Powder™ and Bitter Stop™ liquid for compounding oral suspensions of extremely bitter-tasting drugs [73,159].

## 7. Home Remedies to Minimize the Aversive Taste of Medicines

**Figure 3:**
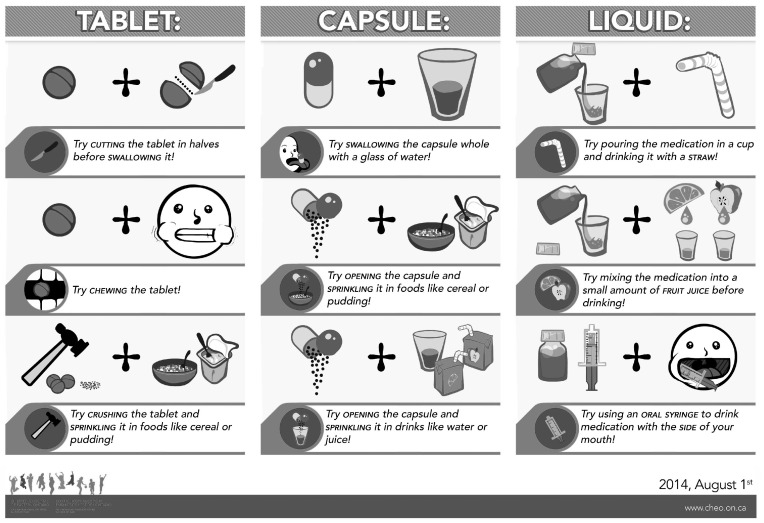
Suggestions for administering medicines to children (adapted from CHEO ED Outreach. Making meds taste better. 2014) [169].

Parents and caregivers faced with a child resistant to taking an unpalatable medicine by mouth have used a range of strategies to make the medicine more acceptable to their children. Some of these strategies are developed by parents and caregivers while others are suggested by healthcare professionals involved in paediatric care (Figure 3). This section summarises common strategies that have been recommended on social media and on hospital and clinical websites.

One recommended strategy is to desensitize the taste buds just before taking a medicine with aversive taste [160]. Desensitization is achieved by sucking on ice, eating ice cream, or brushing teeth and gargling with strong mint-flavoured dental products. These techniques are, however, not acceptable when the medicine is to be taken several times a day or over a prolonged period. Healthcare practitioners have recommended using a straw or oral syringe to administer drugs in liquid forms to the back or side of the mouth to minimize contact with taste receptors on the tongue (Figure 3) [110]. These strategies do not appear to be effective for drugs with strong aversive taste and lingering aftertaste.

A more widely accepted strategy is the co-consumption of a food item to mask the aversive taste of the medicine [110,161]. A wide variety of food items have been suggested, with some recommended to be taken before while others are to be taken together or after the administration of the medicine (Table 2). The reasons underlying these decisions are not apparent in the discussion forums and the effectiveness of these food items in masking the taste of medicines has not been verified with formal human panel taste evaluations. Nonetheless, it may be possible to speculate on the mechanism of taste-masking based on the ingredients discussed in previous paragraphs. Sweeteners with thick textures like honey, maple syrup and jam may render the medicines acceptable by their intense sweetness and pleasant mouthfeel [162,163]. Foods high in fat content, like peanut butter, cheese, and chocolate may reduce drug–taste bud interactions by binding the drug and coating the taste receptors. Fruit juices may increase the medicine acceptance by imparting distinct familiar taste profiles [162] while the chewing of gummies increases saliva production to facilitate a faster eradication of the unpleasant medicine taste in the mouth [164].

## 8. Challenges Associated with Using Taste-Masking Ingredients in Medicines, and Their Responsible Use in Children

Stringent safety requirements impact on the specific strategy that can be adopted in the pharmaceutical industry. Sucrose, because of its cariogenic property [3] and caloric content, is increasingly replaced as a sweetener; however, the non-caloric artificial sweetener replacements are not exempt from causing health issues, and some require warning labels [170]. Salt and fats are, until recently, not used for taste-masking medicines due to safety concerns in some patient groups. For some ingredients, there is a lack of consensus on their safety. Cyclamate, for instance, has been banned in the USA since 1970 but is a permissible artificial sweetener in the EU and Australia. Highly purified stevia is a GRAS ingredient, but crude stevia extracts are not permitted [68]. With excipient safety also dependent on a child’s physiology and some caregivers preferring natural ingredients over synthetically derived ingredients, there are limited excipient options for neonates and infants with immature organ functions [171]. All novel taste-masking ingredients must therefore be shown to be safe for use in children at the dose employed, and the final formulation must be stable during storage, preferably at ambient temperature to increase accessibility to low-resource communities.

There are further concerns that specific taste modifiers can affect drug bioavailability and stability. Amino acids can change the pH of liquid medicines [172], potentially affecting drug solubility and stability. For BCS Class II or Class IV APIs, the use of fatty ingredients or micelle-generating amphiphilic excipients has the potential to substantially alter the in vivo dissolution and absorption of the APIs. In all cases, bioavailability studies must be conducted to establish that drug pharmacokinetics and efficacy are not adversely affected by the adopted taste-masking strategy.

The caregivers’ practice of co-administering food with a drug raises legitimate concerns with regard to food-mediated changes in the pharmacokinetics and pharmacodynamics of the drug. A small amount of food may not cause significant food–drug interactions for most medicines, including those to be given on an empty stomach (e.g., flucloxacillin) [110]. Moreover, when the co-administered food enables a child to willingly take the medicine, a small change in drug bioavailability may be an acceptable compromise compared to having the child refusing to take the medicine at all. However, there are some drugs that should not be administered with certain foods. Drugs metabolised by CYP3A4, including those for treating hypercholesterolemia (simvastatin, atorvastatin), hypertension (nifedipine), and anxiety (buspirone), as well as corticosteroids and antihistamines (fexofenadine) should not be taken with grapefruit [165,173]. Grapefruit contains furanocoumarins which inhibit CYP3A4 and allow more of the intact drug to be absorbed, potentially leading to toxic drug levels in the blood [173]. Dairy products must be used with caution as the calcium in these products can interfere with the absorption and effectiveness of antibiotics (tetracyclines, ciprofloxacin), propranolol, mercaptopurine, nonsteroidal anti-inflammatory drugs, digitalis, amiloride, omeprazole, spironolactone, ranitidine and iron supplements [174].

Breast milk and infant milk formulas should not be used as vehicle for administering medicines with aversive taste to young children dependent on milk as their primary nutrient source. If the medicine taste is not effectively masked, it can cause the child to associate the milk with poor taste and to subsequently refuse to take the milk itself. Some food items are cariogenic and have high caloric content, making them unsuitable as taste modifiers for medicines that are to be taken regularly or on a long-term basis. Honey should not be given to children younger than one year of age due to the risk of infant botulism [110]. Peanut and tree nut butters may trigger allergic reactions and have to be used with caution in child populations with high incidences of nut allergy (e.g., Australia).

## 9. Results and Discussion

The review has shown that there are many common ingredients employed across the food and pharmaceutical industries as taste modifier agents (Table 3). These include sweeteners (natural and artificial), organic acids, flavouring agents, amino acids, and hydrocolloids. There are, however, also differences, e.g., the widespread use of sodium chloride, fats and taste-modifying peptides in the food industry but not in the pharmaceutical industry. The translation of these taste-masking ingredients into medicine formulations will require studies to justify their use, and establishment of safe concentrations in the paediatric population. A key reason underlying these differences is that the food industry can apply complex ingredients and processing methods to generate multiple complex molecules, the identity and concentration of which may not be known, to produce a target taste profile for a food product. Medicines on the other hand are required to use purified materials that meet stringent pharmaceutical excipient quality standards, and to have reproducible critical properties that maintain within- and between-batch consistency of therapeutic efficacy and side effect profiles.

An ideal taste-masking strategy should be simple to implement and generically applicable to a range of oral medicines prescribed across the paediatric age ranges. This has been difficult to achieve due to several factors. Firstly, drug tastes differ widely. A human taste trial of 24 anti-infective liquid medicines has shown products containing the same sweeteners to receive diverse taste scores [35]. Drugs with mildly aversive tastes could be adequately taste-masked using sweeteners, but sweeteners alone were not effective for drugs with strongly aversive tastes or odour. The dosage form design is another factor, with solid preparations more readily taste-masked than solutions containing the same drug in molecular form that can immediately bind with the taste receptors in the mouth to yield stronger perception of the drug taste. Cost effectiveness and scalability are additional factors. Simple techniques using common pharmaceutical excipients are more accessible and economical to implement and scale up, compared to strategies that require novel ingredients and techniques or intermediary steps [175,176]. Further complexities arise when the biopharmaceutical properties like drug dissolution and bioavailability are altered by the taste-masking ingredients [144]. Additionally, the interaction of ingredients is not linear, and mixtures of ingredients in the same taste group can give rise to unexpected taste, as is seen in saccharin and cyclamate [65,66]. Due to these diverse factors, there is currently no generic taste-masking solution that is proven effective across a broad range of drugs and applications. Rather, taste-masking strategies reported in the literature are tailored to specific drug molecules and dosage forms.

Nevertheless, the pharmaceutical industry has come a long way in understanding bitterness perception and evaluation as well as devising methods to reduce the bitterness of medicines for children. This review focuses on ingredients that may be used for taste-masking by simple blending with the API. The two main mechanisms by which the ingredients mask the taste of medicines are either to offer an alternative, more pleasing taste profile or to inhibit the API from interacting with the taste receptor(s). The first mechanism is also used widely and successfully in the food industry but may be difficult to implement in the pharmaceutical industry as it can only use ingredients that meet pharmaceutical standards and methods that do not change the API’s molecular structure. The second mechanism is easier to implement for medicines, as it is achievable through the use of hydrocolloids (and potentially fats) to provide a viscous matrix for immobilizing the drug, the solubilization of drug in micelles of surfactants and polymers, or the formation of drug inclusion complexes with cyclodextrins. Taste-masking is effective by inclusion complex formation only if the drug molecule meets the size and shape requirements of the ring of the cyclodextrin host molecule. This specificity and the requirement for substantial amounts of cyclodextrin to be used for high-dose drugs are limiting factors. Additionally, the drug in free form (not complexed with cyclodextrin) will still interact with taste receptors. Conversely, hydrocolloids, fats and micelle-generating amphiphilic ingredients offer a less selective approach and are applicable to a broader range of drug molecules. They can therefore be used in medicines containing several bitter bioactives.

This review has also revealed the difficulty for an individual ingredient to be effective at suppressing the bitterness of a range of medicines. Although bitter blockers offer huge potential in this regard, a recent review [18] emphasised that more data is required, such as the transiency of effects, before bitter blockers can be used more widely in medicinal products. Moreover, a bitter blocker will only be effective if it binds to the same receptor(s) as the aversive tasting drug molecule, and this can be difficult to achieve given the number of bitter receptors on the tongue and the possibility that a drug may interact with multiple bitter receptors. It is therefore unlikely for a bitter blocker to be universally effective at masking the aversive tastes of a diverse range of drug molecules. More likely, just like the other ingredients reviewed, the bitter blockers will have to be combined with other ingredients offering different taste-masking mechanisms to synergistically achieve effective taste-masking of medicines. This is already evident in the composition of various proprietary pharmaceutical bitter blockers currently available on the market.

While parents and caregivers are passionate about their choice of taste-masking strategy, there are few published studies evaluating the effectiveness of the adopted strategies. Only one study has evaluated the effect of chocolate on the taste of APIs prescribed to children, using 29 healthy young adults. It was found that prior administration of chocolate was effective for masking the bitterness of flucloxacillin and clindamycin solutions, with dark, white and milk chocolates differing in effectiveness [177]. Two other studies were conducted by the food industry, where a Melastomataceae leaf extract native to Indonesia was found to reduce the bitterness of dark chocolate [178], and several cheeses (Baraka cheese of France, Gouda of the Netherlands, Ricotta from Italy, and Brie from France) were found to suppress the bitterness of quinine but not the bitterness of caffeine [129,179]. Quinine interaction with the fatty acids in the cheese was proposed to be the underlying taste-masking mechanism [179]. Based on these limited studies, there is scope to further explore the effectiveness of home remedies as taste blockers for medicines, and to categorise various food items into usability and effectiveness for different age groups in the paediatric population. Potentially, the use of fat-based food items, such as chocolates, soft cheeses and nut butters that provide strong taste and thick viscosity to coat the taste buds have high translational potential. Confirmatory taste trials will allow healthcare professionals to provide much needed evidence-based guidelines to parents and caregivers to manage medicine administration in young children in the home environment.

The preference for sweet taste, and rejection of bitter taste, are well-established in children. While some commercial liquid paediatric medicines have achieved acceptance in children, many paediatric medicines remained poorly accepted despite having sweeteners, flavouring agents and viscosity modifiers as taste modifiers. Medicines with aversive tastes often have poor treatment compliance and outcomes, and low-resource communities appear to bear a disproportionate portion of this burden. As an example, the unpleasant tastes of some oral rehydration supplements used for the treatment of diarrhoea have caused such poor compliance that the refusal of treatment has become life-threatening to the child [180]. Poor treatment outcomes are costly not only to the patient but also to the health services, e.g., poor antimicrobial adherence does not only result in treatment failure for the patient, but subsequent complications may increase the risk of antibiotic resistance in the community. Effective ingredient blends for taste-masking medicines have the potential to provide an affordable and accessible approach for communities with limited resources. More research in this field can be helpful in addressing the needs of children in these communities.

## 10. Conclusions

**Figure 4:**
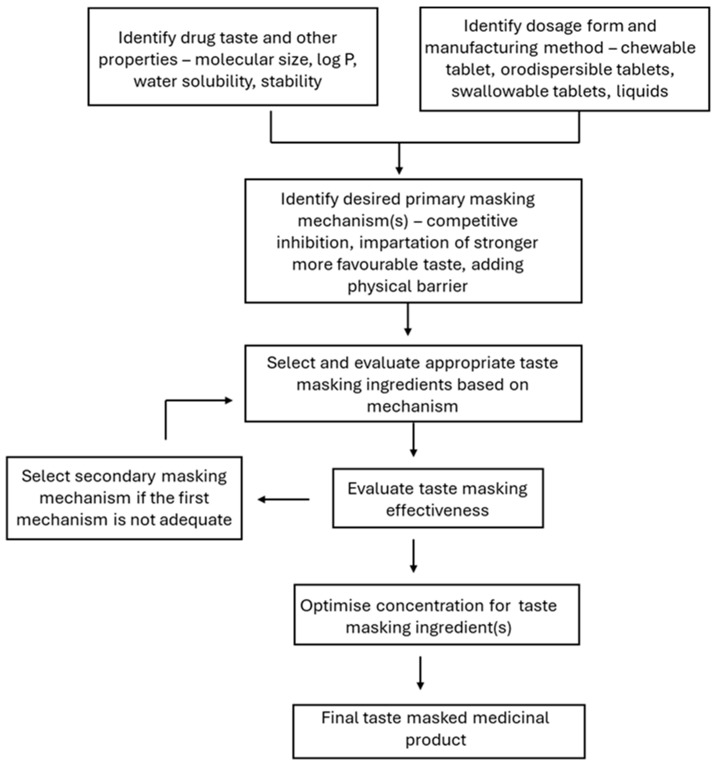
A decision tool to guide the development of simple and effective ingredient-based taste-masking systems for the formulation of palatable paediatric medicines.

A wide range of taste-masking ingredients have been applied in the food and pharmaceutical industries, as well as employed in home settings, to change the taste of food and medicinal products, and these are summarized in Table 3. Sweeteners, flavouring agents, and viscosity enhancers are used in all three areas reviewed. The food industry also uses sodium chloride, taste-modifying peptides, as well as fats (cocoa butter, milk fat), and these may have potential translational opportunities in the pharmaceutical industry. On the basis of this review, a decision tool to guide the development of simple, acceptable, and effective ingredient-based taste-masking systems for drugs with aversive tastes is provided in Figure 4 for the formulation of palatable paediatric medicines.

## Figures and Tables

**Table 1:** Examples of approved paediatric formulations in Australia and the ingredients likely to serve as taste-masking agents.

Brand Name	Drug	Probable ^1^ Taste-Masking Ingredients	Dosage Form
Flucil	Flucloxacillin	Saccharin sodium, sucrose, sodium citrate, ammonium glycyrrhizinate, pineapple flavour, menthol flavour	Oral solution
Flagyl	Metronidazole	Natural soluble lemon flavour, orange oil terpeneless, sucrose	Oral suspension
Zithromax	Azithromycin dihydrate	Sucrose, spray dried artificial cherry, spray dried artificial banana and crema vaniglia flavour	Powder for oral suspension
Predmix	Prednisolone sodium phosphate	Propylene glycol	Oral liquid
Zyrtec	Cetirizine hydrochloride	Betadex, flavour, mannitol, sucralose	Chewable tablet
Zyamis	Midazolam	Saccharin sodium, glycerol, maltitol solution	Oromucosal Solution
Panadol	Paracetamol	Acesulfame potassium, carbomer 934P, flavour, malic acid, maltitol solution, sorbitol solution, sucralose, xanthan gum	Oral suspension
Nurofen Meltlets Citrus	Ibuprofen	Flavour, malic acid, mannitol, microcrystalline cellulose, sucralose	Oral disintegrating tablet

^1^ The term “probable” means the taste-masking ingredients were assumed based on available product information, not directly confirmed by the manufacturer or human taste trial data.

**Table 2:** Food items recommended by caregivers to mask the unpleasant taste of medicines ^1^.

Food Items Used Before Medicine Administration	Food Items That Are Co-Administered with Medicine	Food Items Used Post Medicine Administration
Peanut butter [163]	Yogurt [162]	White grape juice [163]
Maple syrup [163]	Stewed fruit [165]	Jelly beans or a lollipop [163]
Cocoa butter, cashew nut butter or almond nut butter [162]	Maple syrup or jam [165]	Cinnamon gum [164]
	Vegemite [166]	
	Chocolate syrup [163]	
	Vanilla ice cream [163]	
	Melted mint or starburst candy [163]	
	Mashed potatoes [167]	
	Fresh fruit/vegetable juices (pear, grape, carrot, beetroot, ginger, apple, orange, celery) [162]	
	Organic honey [162]	
	Cold and/or citrus flavoured foods/drinks or (sorbets) [162]	
	Pudding or applesauce [166]	
	Chocolate milk [164]	
	Cottage cheese [168]	
	Kool-aid powder [163]	

^1^ While each food item has been verified with at least 2 independent sources, only one reference source has been cited.

**Table 3:** Comparative evaluation of ingredients reviewed in the different categories and applied in the food industry, pharmaceutical industry and home remedies ^1^.

Category of Ingredients	Mechanisms	Food Industry	Pharmaceutical Industry	Home Remedy
Sweeteners	Activate sweet receptor, override other tastes	Sucrose, sugar alcohols, stevia, monk fruit sweetener	Similar to food industry	Honey, maple syrup, sugar, sweet drinks, candy
Salts	Reduce bitter perception and increase sweetness	Sodium chloride, potassium chloride	Sodium chloride has only recently been recommended for compounded medicines	Sodium chloride is not applied per se but salty foods, e.g., vegemite, are used
Acids	Enhance sourness perception and impart specific taste profile	Citric acid, malic acid, acetic acid	Citric acid, malic acid, adipic acid	Vinegar, lemon juice
Peptides and amino acids	Block bitterness receptor and impart more attractive sweetness and umami tastes	Taste peptide, yeast extract	Lysine, glycine	Cheese, yeast-containing products like vegemite
Flavouring agents	Enhance desired taste	Strawberry, vanilla, lemon, mint complex combination of ingredients	Strawberry, bubble gum, cola, lemon	Cinnamon, flavoured drinks and candy
Fats	Induce chemical interactions and provide physical viscous barrier	Cocoa butter, oils, palmitic acids	Phospholipids, cocoa butter	Butter, chocolate, milk, nut spreads, yoghurt
Hydrocolloids	Provide physical viscous barrier, and increase salivation with chewing	Xanthan gum, gelatine, pectin gum, alginate	Xanthan gum, HPMC, gelatine, PEG	Pudding, apple puree
Bitter blockers	Block specific bitter receptors	Taste peptide, yeast extract	Sodium acetate, sodium gluconate	-
Cyclodextrin	Inclusion complex	β Cyclodextrin, γ Cyclodextrin	β Cyclodextrin, γ Cyclodextrin	-

^1^ The most frequently identified ingredients have been compiled. Nonetheless, a wide range of additional substances are employed within both the food and pharmaceutical sectors, whereas home remedies represent complex food products that resist straightforward classification within conventional ingredient taxonomies.

## Data Availability

No new data was created or analysed in this study. Data sharing is not applicable to this article.
